# Nanostructuring silica-iron core–shell particles in a one-step aerosol process†

**DOI:** 10.1039/d4ra01154f

**Published:** 2024-06-06

**Authors:** Delyana Ratnasari, Eka Lutfi Septiani, Asep Bayu Dani Nandiyanto, Kiet Le Anh Cao, Nobuhiro Okuda, Hiroyuki Matsumoto, Tomoyuki Hirano, Takashi Ogi

**Affiliations:** a Chemical Engineering Program, Department of Advanced Science and Engineering, Graduate School of Advanced Science and Engineering, Hiroshima University 1-4-1 Kagamiyama Higashi-Hiroshima Hiroshima 739-8527 Japan ogit@hiroshima-u.ac.jp; b Program Studi Kimia, Fakultas Pendidikan Matematika dan Ilmu Pengetahuan Alam, Universitas Pendidikan Indonesia Jl. Setiabudhi No 229 Bandung West Java 40154 Indonesia; c Materials Research Center, Technology & Intellectual Property HQ, TDK Corporation 570-2 Matsugashita, Minami-Hadori Narita Chiba 286-8588 Japan

## Abstract

Silica-coated iron (Fe@SiO_2_) particles have attracted considerable interest as a potential powder core material due to their distinctive advantages, including higher magnetic saturation and enhanced electrical resistance. In this study, the submicron-sized core–shell Fe@SiO_2_ particles were successfully synthesized in a single step *via* an aerosol process using a spray pyrolysis method assisted by a swirler connector for the first time. Changing the reducing agent concentration (supplied H_2_) and tuning the number of core (Fe) particles were investigated to achieve the desired Fe@SiO_2_ particles. The results indicated that an excessive number of cores led to the appearance of FeO crystals due to insufficient reduction. Conversely, an insufficient number of cores resulted in a thicker SiO_2_ shell, which hindered the penetration of the supplied H_2_ gas. Furthermore, the produced Fe@SiO_2_ particles exhibited soft-ferromagnetic characteristics with an excellent magnetic saturation value of 2.04 T, which is close to the standard theoretical value of 2.15 T. This work contributes new insights into the production of core–shell Fe@SiO_2_ particles, expanding their applicability to advanced soft-magnetic materials.

## Abstract 2

Submicron-sized core–shell Fe@SiO_2_ particles were successfully synthesized using a single-step aerosol technique for the first time.

## Graphical abstract

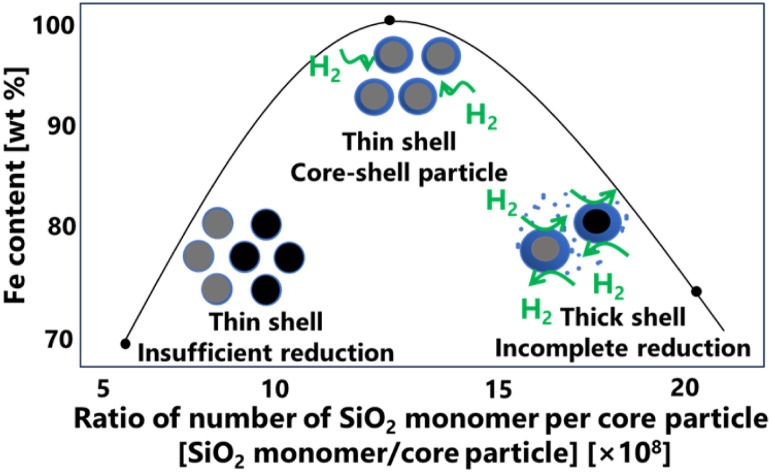

## Introduction

Soft magnetic materials play an important role in electrical and electronic devices such as transformers^1^ and inductors.^2^ As electronic components advance towards higher frequencies, miniaturization, and integration, the demand for soft magnetic materials with high saturation magnetization and low core loss has intensified.^3–7^ Therefore, it is imperative to manufacture suitable materials and structures to meet these requirements. Iron (Fe) stands out as the ideal soft magnetic material for this purpose because of its excellent characteristics (*e.g.*, high saturation magnetization). However, Fe particles are easily oxidized under atmospheric conditions. Moreover, applying Fe particles at high frequencies generates a notable core loss due to the inter-particle eddy current under an alternating magnetic flux, limiting its potential applications. To address these issues, silica-coated iron (Fe@SiO_2_) particles with core–shell structures have been recognized as promising candidates.^8–11^ Fe@SiO_2_ particles play a key role in minimizing eddy current losses by isolating the conductive Fe core from the surrounding environment, thereby enhancing the resistance of the core material to oxidation.^12^ The insulating properties of the SiO_2_ shell prevent the flow of eddy currents inside the core, enhancing the efficiency of devices based on these particles and reducing power dissipation. In addition, core–shell structures with a spherical shape offer distinct advantages over particles with other shapes.^13–15^ This can be explained by the following reasons: (i) the lack of sharp edges in spherical particles minimizes damage to their overall structure, and (ii) spherical core–shell particles enable optimal packing density for the active materials.^16–20^

Numerous research efforts have been dedicated to the development of advanced strategies for synthesizing Fe@SiO_2_ particles in either a multi-step or single-step process. In a multiple-step process, the Fe@SiO_2_ particles with different morphology are synthesized through two or more processes involving Fe particle formation followed by the deposition of SiO_2_ onto the Fe core.^11,12,21–24^ The detailed recent report of these processes is shown in Table S1, ESI.† These processes were performed by combining gas, solid, and liquid phase methods in a relatively long time. Moreover, a complicated process such as particle modification followed by a purification and heating process is required. So far, these methods resulted in non-spherical particles. On the other hand, a single-step process is mainly attempted by gas phase or aerosol methods. The aerosol process has been considered as a more effective and suitable approach for continuous production, holding significant potential for the rational design and synthesis of various functional nanostructured materials with customized composition and morphology.^7,18–36^ Despite its advantages, obtaining pure Fe as a core with a SiO_2_ shell poses challenges as the reduction and coating processes unfold in a single system. The determination of precursor and reduction gas concentrations, along with temperature control, is crucial for successfully obtaining pure Fe as the core.^37,38^ So far, there have been no reports of successfully producing Fe or Fe with SiO_2_ shells in a single aerosol process (see Table S1, ESI†). The common issue in this process when synthesizing Fe particles is the tendency to form oxide phases, such as iron oxides (*e.g.*, Fe_2_O_3_, Fe_3_O_4_), indicating the complexity of synthesizing pure Fe particles.^38,39^ It is considered that higher heat transfer and mass transfer are necessary to prevent the formation of these impurities. Therefore, we propose a novel method for synthesizing Fe@SiO_2_ particles using the swirler connector-assisted spray pyrolysis method. This method provides the distinct advantages of the swirling motion, increasing heat transfer and mass transfer during the reduction process.^40^ Specifically, an improvement of mixing intensity between Fe precursor and reduction gas due to swirling flow could produce Fe without its oxide for the first time. This method is also proven to facilitate the SiO_2_ coating in one-step synthesis during the spray pyrolysis process.^41–43^ Furthermore, particles generated through this method exhibit an adaptable narrow distribution of particle size and have a well-defined spherical shape, which can be adjusted by controlling the spray parameters.

**Fig. 1:**
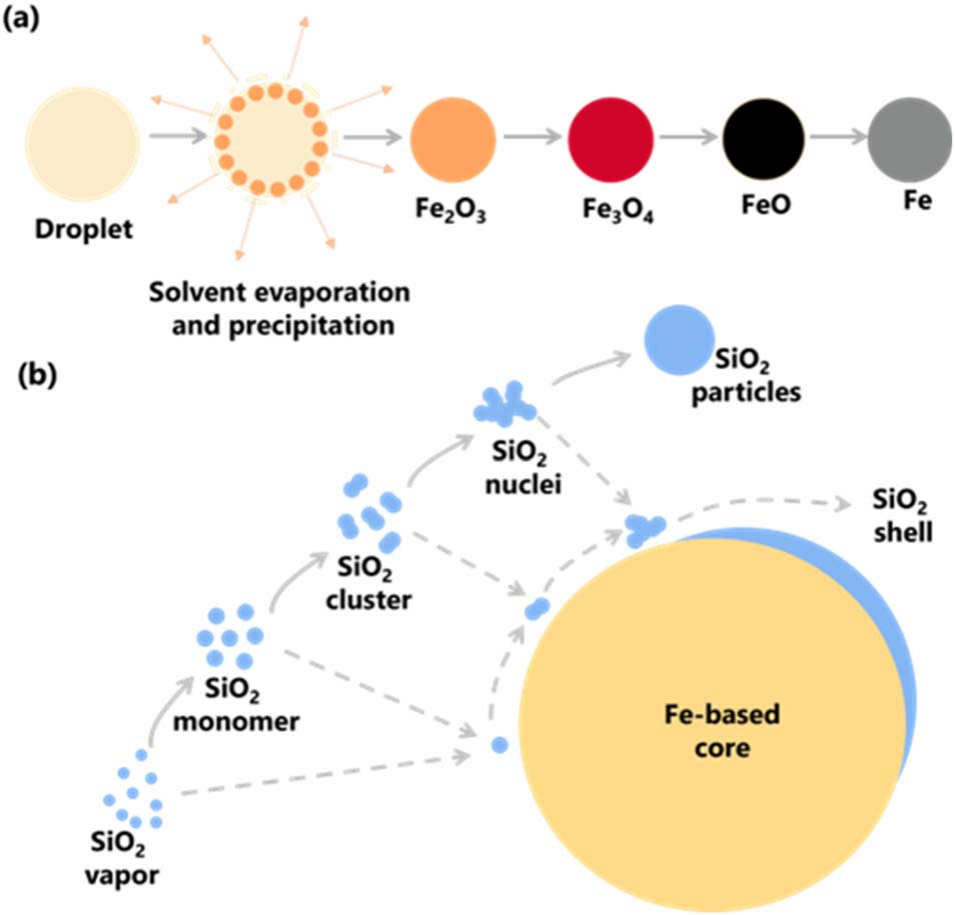
(a) Schematic representation of the synthesis of Fe particles *via* spray pyrolysis route and (b) formation of SiO_2_ on the surface of the Fe-based core.

The schematic representation of synthesizing Fe@SiO_2_ particles produced in this study is presented in Fig. 1(a). In this approach, a precursor dissolved in an aqueous solution was atomized to generate the Fe core in four steps. Firstly, a precursor solution containing Fe ions was atomized, and the droplets thus produced are heated to remove the solvent and decompose it into Fe_2_O_3_.^44^ Secondly, the Fe_2_O_3_ was then reduced to form Fe_3_O_4_,^44^ and further reduction led to FeO.^45^ Finally, the FeO was completely reduced to Fe.^46^Fig. 1(b) also illustrates the proposed formation of SiO_2_ during the coating of the Fe core. The proposed process facilitated the simultaneous formation of Fe (as the core component) and SiO_2_ (as the shell component) from their precursors. During the heating process (see gray arrow), SiO_2_ is formed as monomers, clusters, nuclei, and particles in the gas phase. When a Fe-based core exists, the SiO_2_ is possibly formed onto the core surface following these routes by deposition mechanism. In this deposition, diffusion-adsorption of SiO_2_ monomers on the core surface, attraction and aggregation of SiO_2_ clusters, nuclei, and nanoparticles to the core surface, as well as the growth of nuclei to form SiO_2_ on the core, are possibly occurred (illustrated by gray and dashed arrows in Fig. 1(b)). Based on these routes, the factor involved in successfully synthesizing Fe@SiO_2_ particles was SiO_2_ deposition, depending on the possible collision and interaction mechanism between SiO_2_ components and Fe core surface. This insight suggests a strategic approach to control the ratio of number of SiO_2_ monomer per core particle to achieve successful synthesis of Fe@SiO_2_ particles.

In light of the aforementioned challenges, the objective of this study was to successfully produce submicron-sized core–shell Fe@SiO_2_ particles through a swirler connector-assisted spray pyrolysis by elucidating the influence of the ratio of number of SiO_2_ monomer per core particle on particle synthesis. The number of cores was crucial because it directly impacts the selective deposition and formation of SiO_2_ on the surface of core.^47,48^ In addition, it affected heat transfer phenomena due to the amount of solvent that evaporates from the droplet. Therefore, to investigate the successful formation of Fe@SiO_2_ particles, deliberate variations were made in the number of iron, defined as the number of cores to the carrier gas volume transported during the process. Furthermore, the magnetic properties of the synthesized particles were analyzed in this study. Remarkably, this is the first report of successfully producing one-step submicron core–shell Fe@SiO_2_ particles in the direct aerosol process. Our findings provided new insights into the synthesis of submicron Fe@SiO_2_ core–shell particles and demonstrated their potential for application as advanced soft magnetic materials.

## Experimental

### Materials and methods

**Fig. 2:**
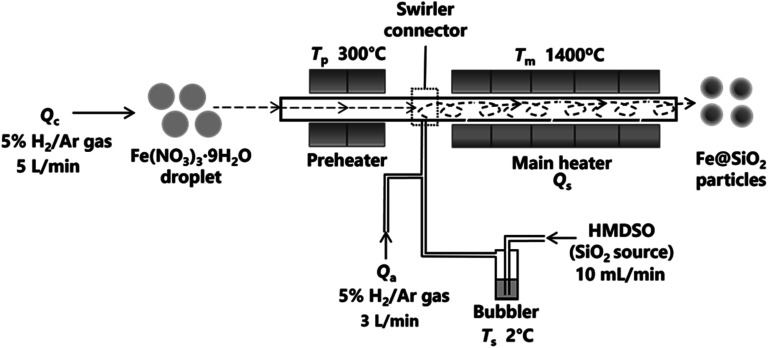
Experimental setup of synthesizing Fe@SiO_2_ particles.

The swirler connector-assisted spray pyrolysis technique was employed to synthesize the Fe@SiO_2_ particles, as shown in Fig. 2. This system consisted of a preheater, swirler connector, and main heater. A precursor solution was prepared by dissolving Fe(NO_3_)_3_·9H_2_O as the Fe source (Fujifilm Wako Pure Chemical Corporation, Osaka, Japan) in ethanol (as reduction agent; 99.8% in purity, Japan Alcohol Corporation, Tokyo, Japan) and ultra-pure water solution with a concentration of 0.10 mol L^−1^. The ethanol concentration varied from 25% to 30% (v/v). To produce spherical particles, the precursor solution was transformed into droplets through an ultrasonic nebulizer (NE-U780, Omron Healthcare Co., Ltd., Kyoto, Japan). Subsequently, these droplets flowed into the preheater with a carrier gas (5% H_2_/Ar) at a flow rate of 5 L min^−1^ (*Q*_c_). In the preheater temperature (*T*_p_) of 300 °C, the precursor droplet was converted to an intermediate product. These intermediate products and hexamethyl disiloxane (HMDSO, the source of SiO_2_) were mixed in the swirler connector. HMDSO vapor was produced from a bubbler at a controllable temperature and was transported into the connector at an HMDSO flow rate (*Q*_s_) of 10 mL min^−1^. An additional carrier gas (*Q*_a_) was introduced into the main heater at a rate of 3 L min^−1^ to ensure the effective mixing of the intermediate product and HMDSO vapor. The *T*_p_ was not varied in this study because an increase in *T*_p_ corresponds to an increase in SiO_2_ formation in the connector. If the SiO_2_ formation increases, it potentially impedes the reduction process.^42,43^ The HMDSO was maintained in a bubbler at a temperature (*T*_s_) of 2 °C. Five electric tubular furnaces were used to maintain a constant temperature (*T*_m_) of 1400 °C in the main heater. The resulting product from the main heater was collected using a bag filter, which was kept at a temperature (*T*_0_) of 135 °C.

In this experiment, the number of cores was varied by changing the nebulization volume level from 1 to 10. To calculate the number of cores, we conducted an additional experiment (see ESI,† Section 2, for details). The experimental conditions are listed in Table 1. The product is labeled as FS-*A-B*, where FS indicates the Fe@SiO_2_ particles, *A* denotes the ethanol volume concentration in the starting solution, and *B* (×10^6^) denotes the number of cores (droplets) per unit volume of the inert carrier gas. For example, FS-25-1.10 represents the Fe@SiO_2_ particles synthesized using 25% (v/v) ethanol with 1.10 × 10^6^ cores per cm^3^ carrier gas.

**Table 1:** Experimental conditions for synthesis of Fe@SiO_2_ particles

Name	Ethanol concentration (%, v/v)	Number of cores (cores per cm^3^ carrier gas)
FS-25-1.10	25	1.10 × 10^6^
FS-25-0.64	25	0.64 × 10^6^
FS-30-0.43	30	0.43 × 10^6^
FS-30-0.54	30	0.54 × 10^6^
FS-30-0.64	30	0.64 × 10^6^
FS-30-0.76	30	0.76 × 10^6^
FS-30-0.99	30	0.99 × 10^6^
FS-30-1.10	30	1.10 × 10^6^

### Material characterization

The morphologies and structures of Fe@SiO_2_ particles were examined through field-emission scanning electron microscopy (FE-SEM; S-5200, 20 kV, Hitachi High-Tech. Corp., Tokyo, Japan) and transmission electron microscopy (TEM; JEM-2010, 200 kV, JEOL Ltd., Tokyo, Japan). The thickness of the shell in all samples was calculated by measuring about 100 randomly chosen particles from TEM images. The particle size distribution was determined using ImageJ software,^49^ which analyzed approximately 300 particles in the SEM images. X-ray diffraction (XRD) patterns of the particles were identified using XRD equipment (D2 PHASER, Bruker Corp., Billerica, MA, USA). To determine the mass fraction of crystalline phases, a quantitative analysis was conducted using Rietveld analysis with TOPAS software.^50^ The cross-sectional image of Fe@SiO_2_ particles was investigated using a TEM (JEM-2100F, 200 kV, JEOL Ltd., Tokyo, Japan), and the elemental distribution was analyzed through energy-dispersive X-ray spectroscopy (EDS) to map Fe, Si, and O. Epoxy resin was used to embed the Fe@SiO_2_ particles in a grid for these observations. Subsequently, the particle-epoxy mixture was cured and processed into thin specimens using ion milling (PIPS, GATAN Inc., Pleasanton, CA, USA). TEM images were acquired following the ion-milling procedure. The magnetic properties of the Fe@SiO_2_ particles were evaluated *via* vibrating sample magnetometry (VSM) within a ±20 kOe field, employing a high-sensitivity VSM System TM-VSM311483-HGC (Tamakawa Co., Ltd.).

## Results and discussion

### Effect of changing the ethanol volume concentrations and number of cores on the formation of Fe@SiO_2_ particles

**Fig. 3:**
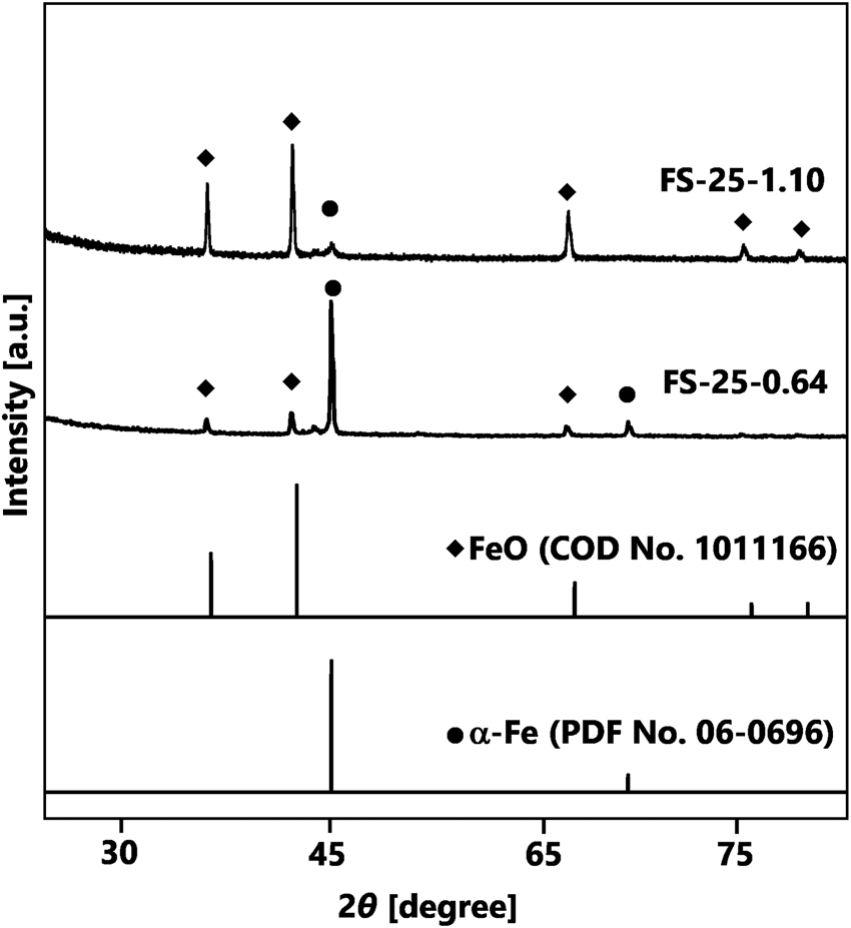
XRD patterns of samples prepared using 25% (v/v) ethanol with different number of cores.

The first strategy for synthesising Fe@SiO_2_ particles was carried out by controlling the number of cores in the synthesis process, using 25% (v/v) of ethanol as the reduction agent. Fig. 3 represents the crystal structures of the Fe@SiO_2_ particles produced with different numbers of cores. It is evident that α-Fe and FeO were identified in all samples according to their PDF No. 06-0696 and COD No. 1011166, respectively. Notably, reducing the number of cores significantly improved the α-Fe peaks while decreasing the FeO peaks. Quantitatively, the Rietveld analysis revealed that the reduction in the number of cores resulted in an increase in the mass percentage of α-Fe from 13% to 56%. Although decreasing the number of cores led to an enhancement in the reduction efficiency, the complete conversion of FeO to α-Fe was still inadequate. Therefore, a further strategy was employed, whereby the degree of reduction was increased using a higher volumetric ethanol concentration under atmospheric conditions.

In contrast to the lower degree of reduction achieved with 25% (v/v) ethanol, the use of 30% (v/v) ethanol with 1.10 × 10^6^ cores per cm^3^ carrier gas significantly enhanced the α-Fe peak and reduced the FeO peak, as shown in Fig. S2 (ESI†). The increase in the degree of reduction with 30% (v/v) ethanol was attributed to an increase in the generation of H_2_ gas, as indicated in reaction (1):^25^

1 C_2_H_5_OH + 3H_2_O → 2CO_2_ + 6H_2_

**Fig. 4:**
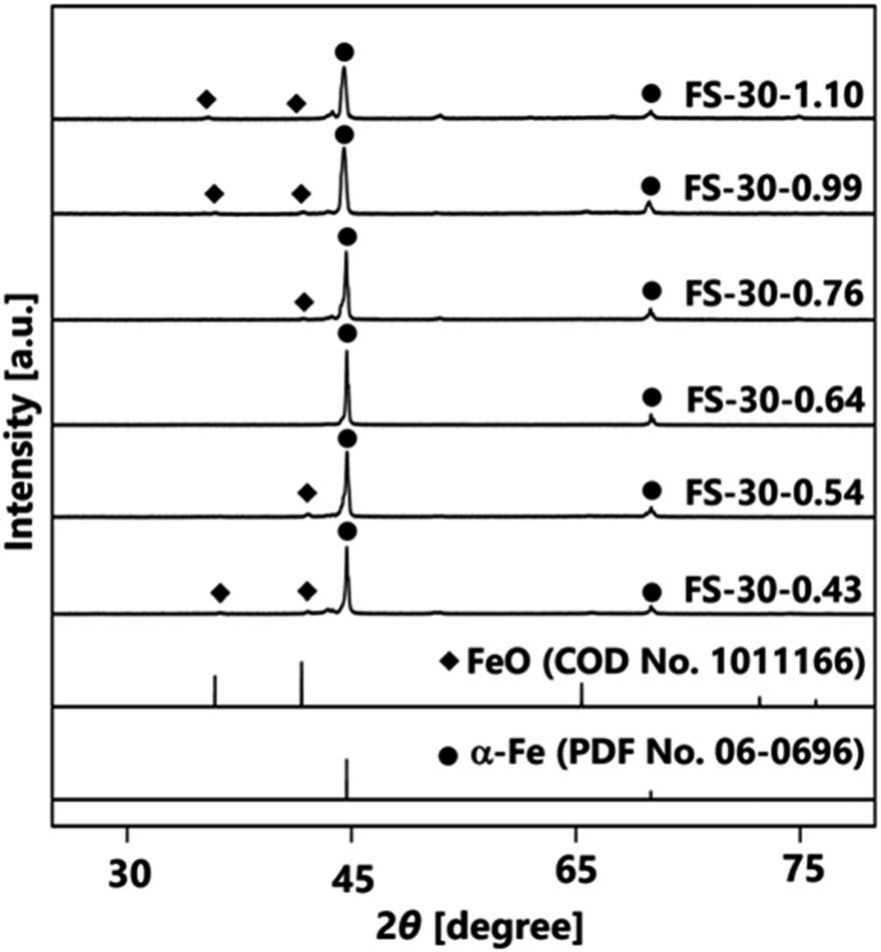
XRD patterns of samples prepared using 30% (v/v) of ethanol with different number of cores.

Further investigation was performed by varying the number of cores from 1.10 × 10^6^ to 0.43 × 10^6^ cores per cm^3^ carrier gas. The XRD patterns in Fig. 4 illustrate the effects of different numbers of cores on the crystal structures of Fe@SiO_2_ particles. The synthesized particles with a higher number of cores, specifically in the range from 1.10 × 10^6^ to 0.76 × 10^6^ cores per cm^3^ carrier gas (FS-30-1.10 to FS-30.0.76), exhibited the crystal structure of α-Fe and FeO. Meanwhile, as the number of cores decreased from 0.76 × 10^6^ to 0.64 × 10^6^ cores per cm^3^ carrier gas, the crystal structure of the synthesized particles reflected the crystal structure of α-Fe without FeO, as shown in the XRD pattern of the FS-30-0.64 sample. Peaks were detected at 44.6° and 65.0°, which corresponded to the bcc crystal plane of α-Fe (PDF No. 06-0696). Notably, a further decrease in the number of cores resulted in FeO crystal formation, as observed in the XRD patterns of samples, FS-30-0.54 and FS-30-0.43. These results indicate the importance of precisely controlling the number of cores to obtain Fe@SiO_2_ particles without the undesired formation of FeO.

**Fig. 5:**
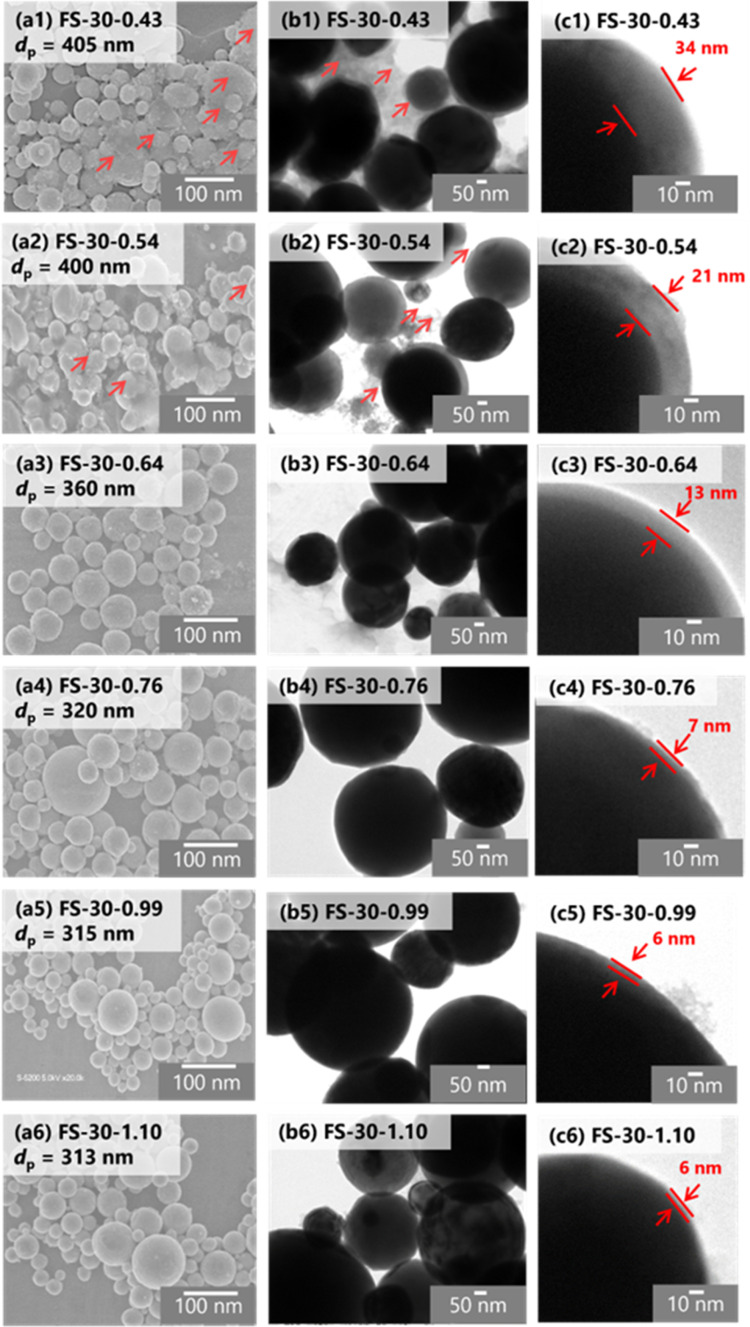
The effect of the number of cores on the morphology of Fe@SiO_2_ particles. (a) SEM and TEM images of Fe@SiO_2_ particles at a different number of cores: (1) 0.43 × 10^6^, (2) 0.54 × 10^6^, (3) 0.64 × 10^6^, (4) 0.76 × 10^6^, (5) 0.99 × 10^6^ and (6) 1.10 × 10^6^ cores per cm^3^ carrier gas at (b) low magnification and (c) high magnification.

Furthermore, the morphology and structure of the Fe@SiO_2_ particles synthesized with varying number of cores were observed using SEM and TEM images, as shown in Fig. 5(a–c). SEM images of all the samples revealed spherical submicron particles, as shown in Fig. 5(a), indicating particle sizes ranging from 313 to 405 nm. The SEM images of FS-30-0.43 and FS-30-0.54 samples shows the presence of free SiO_2_ nanoparticles (indicated by the red arrow in Fig. 5(a1 and a2)). The TEM images (Fig. 5(b and c)) revealed the particles with a core–shell structure with different shell thicknesses. By increasing the number of cores from 0.43 × 10^6^ to 1.10 × 10^6^ cores per cm^3^ carrier gas, the shell thickness decreased from 34 to 6 nm. The generation of free SiO_2_ nanoparticles was also confirmed by TEM images (indicated by the red arrow in Fig. 5(b1 and b2)) with a thicker shell thickness (Fig. 5(c1 and c2)). These observations indicated that an insufficient number of cores allowed SiO_2_ clusters to easily accumulate on the Fe core, as the higher the SiO_2_ concentration surrounding the Fe particles led to a thicker SiO_2_ shell.^43^ As the number of cores increased, free SiO_2_ nanoparticles were not observed in either SEM or TEM images, as shown in Fig. 5(a3–a6 and b3–b6). In addition, high magnification of the TEM images revealed a decrease in shell thickness from 13 to 6 nm with an increase in the number of cores (see Fig. 5(c3–c6)). However, the shell thickness did not change significantly in the samples FS-30-0.76, FS-30-0.99, and FS-30-1.10, indicating an excessive number of cores. The excessive number of cores led to the formation of a thinner SiO_2_ shell.

**Fig. 6:**
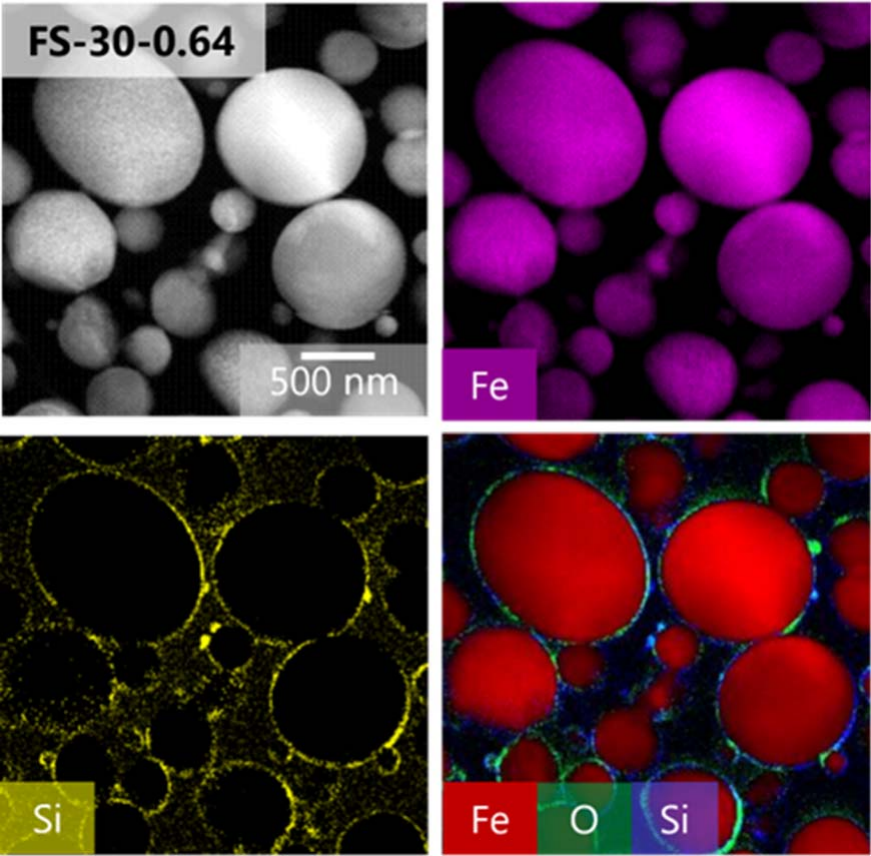
TEM image and elemental mapping of the cross-section of Fe@SiO_2_ particles (FS-30-0.64).

A cross-sectional TEM analysis was conducted to further verify the structure of the synthesized particles in the FS-30-0.64 sample. The successful synthesis of the core–shell structure of the Fe@SiO_2_ particles was confirmed by EDS mapping of the cross sections of the particles, as presented in Fig. 6, which indicated the presence of Fe, Si, and O atoms throughout the particles. The presence of O and Si atoms on the surface of the Fe core suggested that it was completely covered by the SiO_2_ shell.

**Fig. 7:**
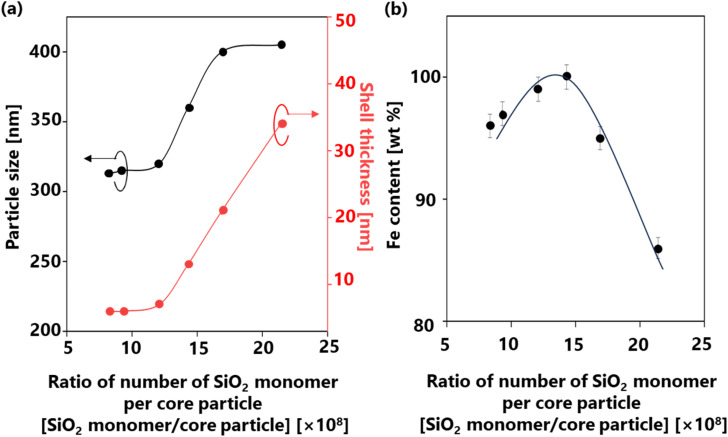
Correlation between the number of SiO_2_ monomer per core particle ratios on the (a) particle size and shell thickness and (b) α-Fe content in the Fe@SiO_2_ particles.

Controlling the number of cores affected the ratio of the supplied SiO_2_ per core particle. The supplied SiO_2_ was defined by the number of SiO_2_ monomers that can possibly generated. Therefore, the relationship between the number of SiO_2_ monomer per core particle ratios on several parameters (*e.g.*, particle size, shell thickness, and α-Fe content) is illustrated in Fig. 7 to confirm the phenomenon during the synthesis. The same number of SiO_2_ monomers was used to calculate this ratio, given that a consistent amount of SiO_2_ was supplied to the system. Meanwhile, the number of core particles varied from 0.43 × 10^6^ to 1.10 × 10^6^ cores per cm^3^ carrier gas, assuming that all droplets were converted to particles. Detailed calculations of the number of SiO_2_ monomer per core particle ratios are provided in ESI,† Section 4 and the results are presented in Table 2. Fig. 7(a) depicts the relationship between the number of SiO_2_ monomer per core particle ratio on particle size and shell thickness. A decrease in the number of cores led to an increase in the number of SiO_2_ monomer per core particle ratio, resulting in larger particle sizes ranging from 313 to 405 nm (depicted by the black line). Assuming that each droplet was transformed into one particle in conventional spray pyrolysis, the number of initial droplets is identical to the number of particle synthesized.^51^ Therefore, the change in the final particle size is attributed to the increase in the shell thickness from 6 to 34 nm (indicated by the red line).

**Table 2:** The ratio of number of SiO_2_ monomer per core particle

Name	Number of cores [cores per cm^3^ carrier gas]	Ratio of number of SiO_2_ monomer per core particle [SiO_2_ monomer/core particle]
FS-30-0.43	0.43 × 10^6^	21.4 × 10^8^
FS-30-0.54	0.54 × 10^6^	16.9 × 10^8^
FS-30-0.64	0.64 × 10^6^	14.3 × 10^8^
FS-30-0.76	0.76 × 10^6^	12.1 × 10^8^
FS-30-0.99	0.99 × 10^6^	9.32 × 10^8^
FS-30-1.10	1.10 × 10^6^	8.35 × 10^8^

The number of cores directly influences the space available for deposition and growth of SiO_2_; a large number of cores leads to thinner shells due to SiO_2_ insufficiency.^52^Fig. 7(b) highlights the significant impact of the number of SiO_2_ monomer per core particle ratio on the α-Fe content. A high mass percentage (>80 wt%) of α-Fe was detected in all samples, indicating the quality of our synthesis method. The detailed of α-Fe and FeO content are shown in Table S3 (ESI†). When the number of cores decreased from 1.10 × 10^6^ to 0.76 × 10^6^ cores per cm^3^ carrier gas, the ratio of the number of SiO_2_ monomer per core particle increased from 8.35 × 10^8^ to 12.1 × 10^8^ SiO_2_ monomer/core particle. Consequently, the α-Fe content increased from 96 wt% (FS-30-1.10 sample) to 99 wt% (FS-30-0.76 sample). This revealed that a low number of SiO_2_ monomer per core particle ratio in this case leads to incomplete reduction due to insufficient atmospheric reduction. As the number of cores decreased to 0.64 × 10^6^ cores per cm^3^ carrier gas, the ratio of the number of SiO_2_ monomer per core particle increased to 14.3 × 10^8^ SiO_2_ monomer/core particle, enabling the attainment of 100 wt% α-Fe content. Conversely, the continued increase in the number of SiO_2_ monomer per core particle ratio from 14.3 × 10^8^ to 21.4 × 10^8^ SiO_2_ monomer/core particle resulted in a decrease in the α-Fe content from 100 wt% (FS-30.0.64) to 86 wt% (FS-30-0.43). This decrease is attributed to an excessively high ratio of SiO_2_ monomers to the number of cores, which hinders heat transfer and H_2_ gas penetration due to the formation of thick shells.^53^

### Proposed mechanism for the formation of SiO_2_-coated Fe with different number of cores

**Fig. 8:**
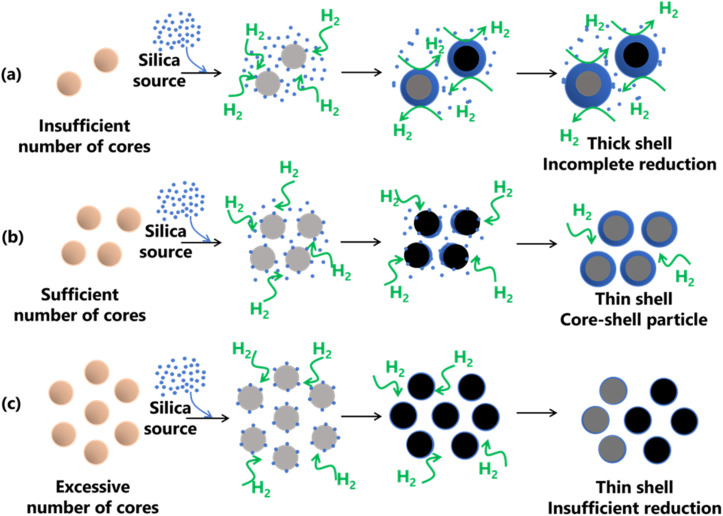
Proposed mechanism for the formation of SiO_2_-coated Fe with (a) insufficient, (b) sufficient, and (c) excessive number of cores.

The formation of Fe@SiO_2_ particles was successfully achieved by carefully regulating the interaction between the Fe core and SiO_2_ components, as shown in Fig. 8. Throughout the synthesis process, the process conditions were kept constant. The only variation introduced was the number of cores entering the preheater. After leaving the preheater, an intermediate product was generated and mixed with the SiO_2_ source in the connector. They proceeded to the main heater, where Fe and SiO_2_ were converted from their raw precursors, as shown in Fig. 1. The SiO_2_ monomer underwent nucleation and growth by consuming other SiO_2_ monomers, eventually forming clusters. The cluster represents the minimum requirement for SiO_2_ to be attracted to the surface of the Fe-based core *via* heterogeneous nucleation. When SiO_2_ monomers interacted with the core in the main heater, three routes were possible, as shown in Fig. 8(a–c). These routes have been marked to have insufficient (samples FS-30-0.43 and FS-30-0.56), sufficient (sample FS-30-0.64), and an excessive number of cores (samples FS-30-0.76, FS-30-0.99, and FS-30-1.10). Fig. 8(a) illustrates the proposed mechanisms associated with an insufficient number of cores. SiO_2_ clusters are prone to being readily scavenged on the Fe core, leading to the formation of thicker shells. However, when the number of available cores is low, the SiO_2_ monomers tend to undergo homogeneous nucleation, resulting in the generation of free SiO_2_ nanoparticles.^54^ Furthermore, according to Table S4 (ESI†), the mole concentration of supplied H_2_ per mole core particle ratio in the FS-30-0.43 and FS-30-0.54 samples were 200 and 157 moles of H_2_/moles of core particles, respectively. These data indicate that despite the insufficient number of core particles, the mole concentration of supplied H_2_ per core particle remained high.

Nevertheless, the reduction process was incomplete because of the presence of FeO in the resulting product. This is likely due to the excessively thick shells hindering heat transfer to the core and impeding the penetration of H_2_ gas, which impeded the reduction process. Fig. 8(b) shows the proposed mechanism when a sufficient number of cores were available. In this case, sufficient ratio between SiO_2_ monomer per core particle results in the complete formation of core–shell particles. This sufficient ratio also promotes heterogeneous nucleation, predominantly favoring SiO_2_ formation on the surface of the core, leading to the creation of a thinner shell. In addition, as indicated in Table S4 (ESI†), the sufficient mole concentration of supplied H_2_ per mole core particles ratio is 133 moles of H_2_/moles core particles. This ratio indicates the amount of supplied H_2_ to complete the reduction process of FeO to α-Fe particles. Fig. 8(c) illustrates the proposed mechanism for an excessive number of cores, which allowed thin shell formation due to insufficient reduction. As shown in Table S4 (ESI†), the mole concentration of supplied H_2_ per mole core particles ratio for samples FS-30-0.76, FS-30-0.99, and FS-30-1.10 is 113, 87, and 78 moles of H_2_/moles core particles, respectively. These data indicate that the mole concentration of supplied H_2_ per mole core particles was lower compared to the case with a sufficient number of cores. This is attributed to the limitations in the reduction process, resulting in the insufficient reduction of FeO to α-Fe particles (confirmed by the XRD pattern in Fig. 4).

### Magnetic characteristics of the synthesized Fe@SiO_2_ particles

**Fig. 9:**
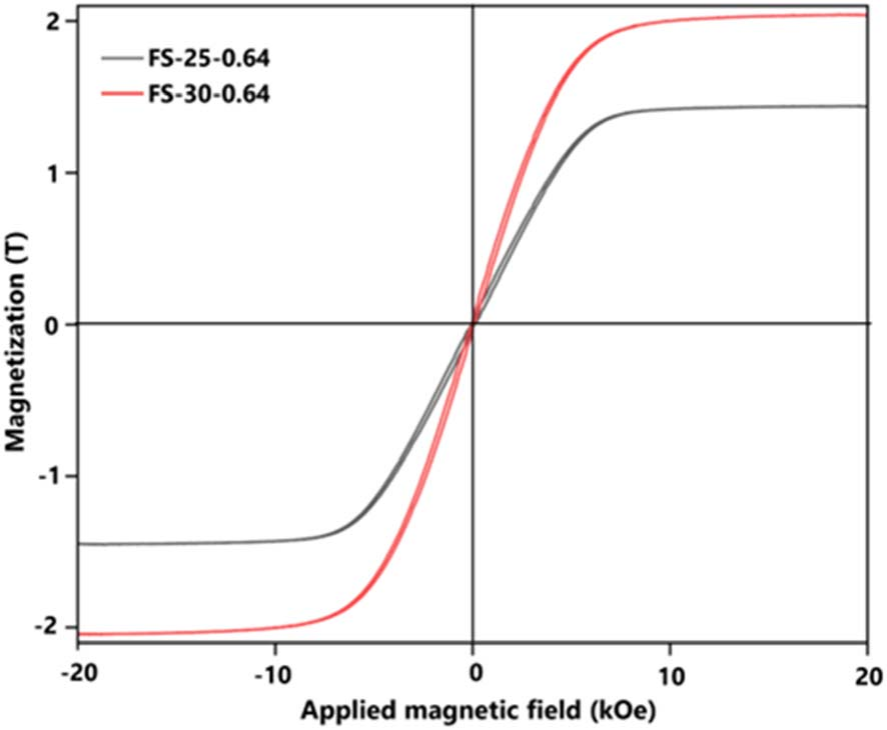
Magnetic hysteresis loop values of prepared Fe@SiO_2_ particles from samples FS-25-0.64 and FS-30-0.64.

To emphasize the successful production of Fe@SiO_2_ particles in this novel approach, the magnetization characteristics (*M*_s_) value of the Fe@SiO_2_ particles were confirmed. Fig. 9 shows the hysteresis loops for the measured SiO_2_-coated Fe samples at different ethanol volume concentrations. The graph shows the characteristics of a soft magnetic material with a high magnetization value. It possessed a high saturation magnetization (more than 1.8 T), which is close to the standard theoretical value of 2.15 T.^55,56^ Adjusting the ethanol volume concentration with a constant number of cores (0.64 × 10^6^ cores per cm^3^ carrier gas) from 25% to 30% (v/v) resulted in increased *M*_s_ values from 1.87 to 2.04 T. The ethanol volume concentration acts as a reduction agent, facilitating sufficient atmospheric reduction (confirmed by the XRD pattern in Fig. S3 in the ESI†). In the FS-25-0.64 sample, the synthesized particles retained FeO, while in the FS-30-0.64 sample, FeO was not detected. It can be concluded that FeO negatively impacts magnetic performance, and the successful transformation of FeO into α-Fe within the product leads to enhanced magnetic properties. Notably, FS-30-0.64 with the *M*_s_ value of 2.04 T is close to the theoretical value of Fe particle's *M*_s_. This slightly different value may be attributed to the presence of SiO_2_ shell.^21^ Based on the *M*_s_ value, it is worth noting that the Fe@SiO_2_ particles produced through our method show promise for practical use, particularly in electronic components like powder core inductors. This finding is fundamental for this component development with a significant prospect. In future work, the application of the synthesized Fe@SiO_2_ particles in a powder core will be investigated by examining its DC bias characteristics, packing density, and eddy current loss value.

## Conclusions

In this study, the spherical and submicron Fe@SiO_2_ particles were successfully synthesized in a single step, marking a significant advancement in material synthesis. The synthesis of Fe@SiO_2_ particles was achieved by precisely adjusting the concentration of ethanol as the reducing agent and controlling the number of core (Fe) particles during synthesis. At the same number of cores, increasing the ethanol concentration from 25% to 30% (v/v) led to the more successful reduction of FeO to Fe due to improved H_2_ gas generation from the ethanol. Furthermore, at the ethanol concentration of 30% (v/v), comparative analysis from different numbers of core particles revealed that this parameter remarkably influenced the successful production of Fe@SiO_2_ particles. The number of cores contributed to the delicate balance between core and reduction gas, as well as Fe core and SiO_2_ monomers. This investigation revealed that the successful production of Fe@SiO_2_ particles is obtained by applying a sufficient number of cores, 0.64 × 10^6^ cores per cm^3^ carrier gas. When the number of cores is too excessive or insufficient, the FeO is detected in the synthesized Fe@SiO_2_ particles. An excess number of cores yielded Fe@SiO_2_ particles with thin shells and residual FeO due to incomplete reduction, while insufficient cores led to thicker SiO_2_ coatings and undesired FeO formation, attributed to hindered heat transfer and limited H_2_ gas penetration. Furthermore, the produced Fe@SiO_2_ particles exhibited a soft-ferromagnetic characteristic with a magnetic saturation value approaching the standard theoretical value, indicating promising properties for various applications. Our findings present a novel material development strategy to address the needs of advanced technologies, particularly in soft magnetic materials. This study contributes valuable insights for future research and technological innovation in material science.

## Author contributions

Delyana Ratnasari: writing – original draft, writing – review & editing, investigation, formal analysis; Eka Lutfi Septiani: writing – review & editing, investigation, formal analysis; Asep Bayu Dani Nandiyanto: writing – review & editing; Kiet Le Anh Cao: writing – review & editing; Nobuhiro Okuda: writing – review & editing, investigation, conceptualization; Hiroyuki Matsumoto: writing – review & editing, investigation, conceptualization; Tomoyuki Hirano: methodology, validation; Takashi Ogi: writing – review & editing, funding acquisition, visualization, conceptualization, supervision.

## Conflicts of interest

There are no conflicts to declare.

## Supplementary Material

RA-014-D4RA01154F-s001
