# Supplementary material for: Nanostructuring silica-iron core–shell particles in a one-step aerosol process
Source: RSC Adv. 2024 Jun 6;14(26):18171–80. doi: 10.1039/d4ra01154f (PMC11155725; doi:10.1039/d4ra01154f)
Supplement: RA-014-D4RA01154F-s001 [file RA-014-D4RA01154F-s001.pdf]

Electronic Supplementary Material (ESI) for RSC Advances.
This journal is © The Royal Society of Chemistry 2024

Electronic Supplementary Information

Nanostructuring Silica-iron Core-shell Particles in a One-step Aerosol Process

Delyana Ratnasari,[a] Eka Lutfi Septiani,[a] Asep Bayu Dani Nandiyanto,[b] Kiet Le Anh Cao,[a] Nobuhiro Okuda,[c] Hiroyuki Matsumoto,[c] Tomoyuki Hirano,[a] and Takashi Ogi [a†]

[a]Chemical Engineering Program, Department of Advanced Science and Engineering, Graduate School of Advanced Science and Engineering, Hiroshima University, 1-4-1 Kagamiyama, Higashi-Hiroshima, Hiroshima 739-8527, Japan

[b]Program Studi Kimia, Fakultas Pendidikan Matematika dan Ilmu Pengetahuan Alam, Universitas Pendidikan Indonesia, Jl. Setiabudhi No 229, Bandung, 40154, West Java, Indonesia

[c]Materials Research Center, Technology & Intellectual Property HQ, TDK Corporation, 570-2 Matsugashita, Minami-Hadori, Narita, Chiba 286-8588, Japan

[†]Corresponding author: Takashi Ogi, ogit@hiroshima-u.ac.jp

Tel/Fax: +81-82-424-3765

Contents

1. **Recent Report of $Fe@SiO_2$ Synthesis on Last Decades** S-3

2. **Experiment for Calculating the Number of Cores** S-5

3. **Effect of Increasing the Degree of Reduction with an Excessive Number of Cores on the Crystal Structure** S-8

4. **Determining the Ratio of Number of SiO_2 Monomer per Core Particle** S-9

5. **Rietveld Refinement Result of $Fe@SiO_2$ Particle at Different Number of Core**S-11

6. **Calculation of the Ratio of Mole of Supplied H_2 per Mole Core Particle**.......... S-12

7. **Effect of Different Degrees of Reduction with Sufficient Number of Cores in the Crystal Structure** S-14

References... S-15

1. Recent Report of $Fe@SiO_2$ Synthesis on Last Decades

To highlight our novel approach in synthesizing $Fe@SiO_2$ particles, we incorporate a comprehensive literature review from the recent reports. The classification of this review is structured around the varying processes to successfully produce $Fe@SiO_2$ particles, categorized as either a multi-step or single-step process, as shown in **Table S1**.

Table S1. A recent report of $Fe@SiO_2$ synthesis in the last decades

Number of syntheses	Method and its condition	Raw material	Final product	Finding and remark
Multi-step synthesis	Hydrothermal reaction and Stöber method with total synthesis of 42 h	$FeCl_3 \cdot 6H_2O$ as Fe source and TEOS as SiO_2 source	Nanocapsules of $Fe@SiO_2$ with 10-35 nm shell thickness	$Fe@SiO_2$ with multicore Fe is obtained through 4-step synthesis, i.e., β-FeOOH, β-FeOOH@SiO_2, α-Fe_2O_3@SiO_2, and $Fe@SiO_2$. The production of Fe@ in this process involves a complex and time-consuming reaction.[1]
Multi-step synthesis	Hydrothermal reaction and the Stöber method. This process requires 18 hours	Iron (II) sulfate as Fe source and TEOS as SiO_2 source.	$Fe@SiO_2$ particles are cubic in shape, with a size of 500 nm and a shell thickness ranging from 10 to 20 nm.	Fe particles can be obtained by the hydrothermal reaction. It is found that a small amount of the residue of surfactant (ethylenediamine) helps the successful SiO_2 coating on the Fe particle. This process study underscores the need for surfactants to modify Fe particle's surface. [2]
Multi-step synthesis	Co-precipitation and Stöber method. The total duration for synthesis is 24 hours.	$Fe(NO_3)_3 \cdot 9H_2O$ as Fe source and TEOS as SiO_2 source.	$Fe@SiO_2$ with chain-like formation. The particle size is 13 nm with 4 nm shell thickness.	$Fe@SiO_2$ containing Fe_2O_3 was produced. Oleic and citric acid were used as surface-capping agents to assist the coating process. This process highlights the surface modification of cores and may need additional processes to reduce Fe_2O_3 to Fe.[3]
Multi-step synthesis	Mechanical milling and Stöber method. The complete synthesis process	Fe Powder and TEOS as SiO_2 sources.	Flake-shape of $Fe@SiO_2$ with particle size ranging from 5-15 μm and 1-2	This process used Fe powder to synthesize Fe flakes. The mineral spirit was then added to the powder mixture to avoid oxidation during milling. It is noteworthy Fe powder is used as

	requires 6 hours.		μm shell thickness	raw material, which is less abundance. [4]
Multi-step synthesis	Hydrothermal reaction and Stöber method and required 18 hours process.	$FeCl_3 \cdot 6H_2O$ as Fe source and TEOS as SiO_2 source.	Fe@SiO_2 with asymmetric shape. The measured particle size is 275 nm with 13 nm shell thickness.	Fe@SiO_2 can be synthesized without additional surfactant. However, the particle shape was irregular. This process cannot be used for obtaining spherical particle.[5]
One-step synthesis	Aerosol spray pyrolysis.	$Fe(NO_3)_3 \cdot 9H_2O$ and $FeCl_3 \cdot 6H_2O$ as Fe source.	FeO with dense structure and submicron size of the particle.	FeC, Fe, and FeO are formed solely in spray pyrolysis. A post-treatment using 100% H_2 gas did not effectively produce Fe without FeO. This finding suggested that a higher temperature and mass transfer of the solid and reduction gas are necessary. [6]
One-step synthesis	The hydrogen reduction-assisted ultrasonic spray pyrolysis with 2 hours operating time	$Fe(NO_3)_3 \cdot 9H_2O$ as Fe source.	Si-dopped Fe_3O_4, Si-dopped-FeO and Fe.	The composite particle of Fe, FeO, Fe_3O_4, and Fe_2SiO_4 is obtained. An additional process is needed to reduce the FeO state to Fe.[7]

2. Experiment for Calculating the Number of Cores

2.1 Experimental Setup for the Calculation of the Number of Cores

The number of cores is the most important factor in the synthesis of Fe@SiO_2 particles. Therefore, to understand the effect of the number of cores, we conducted a simple experiment as shown in **Fig. S1**. In this experiment, we attempted to collect the atomized droplets for 30 minutes. A precursor solution containing 0.1 mol/L $Fe(NO_3)_3.9H_2O$, ethanol 30% (v/v), and ultra-pure water was used as the solvent. First, the precursor was altered by an ultrasonic nebulizer into droplets, which were carried by 5% H_2/Ar gas as a carrier gas flow rate (Q_c) of 5 L/min. In addition, atomized droplets were condensed using a condenser. The condensed droplets were collected in a flask. Their volume was assumed to be an atomized precursors.

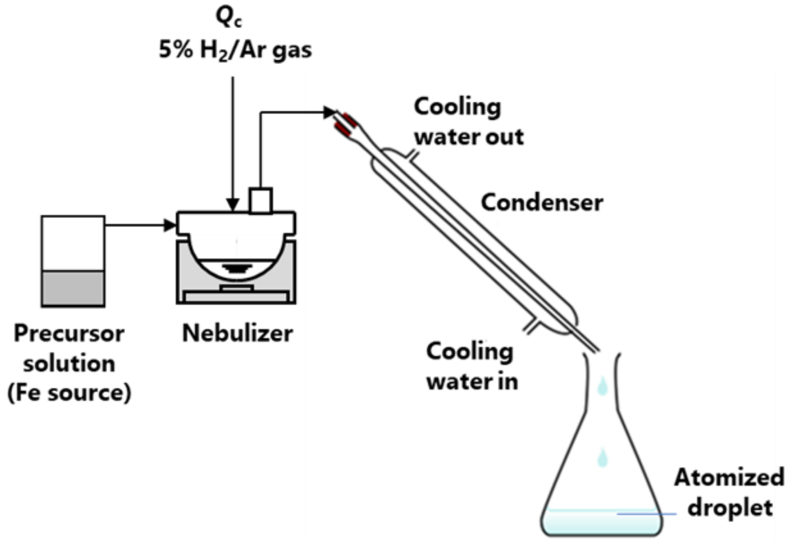

Fig. S1 Experimental setup for calculation of the number of cores.

2.2 Calculating the Experimental Number of Cores per cm^3 Carrier Gas

According to the manufacturer's guidelines, the duty cycle of an ultrasonic nebulizer can be controlled by changing the nebulization volume. The ultrasonic nebulizer can change the nebulization volume from 1 to 10, which represents a linear duty cycle from 30% to 100%. The duty cycle represents the ratio of active nebulization time to total treatment time, which is related to the number of cores produced by the nebulizer. Therefore, the number of cores in different levels of nebulization volume can be calculated by predicting the atomization rate at the maximum duty cycle using the experimental setup in **Fig. S1**. The atomization rate at the maximum duty cycle (i.e., nebulization volume of 10) was 0.00972 mL/s. When the nebulization volumes were 2, 3, 5, and 7, the atomization rate was calculated by multiplying the duty cycle at each levels by the atomization rate at the maximum duty cycle. The number of cores per cm^3 carrier gas ($C_{n,w}$) was then calculated using the equation (1):[8]

$$C_{n,w} = \frac{Q_w}{V_d Q_g} \qquad (1)$$

where Q_w is the precursors rate, V_d is the volume of droplets, and Q_g is the volume of the carrier gas. The droplet is assumed to be spherical with a droplet diameter (d_d) of 4.6 μm[9] according to the specifications of the ultrasonic nebulizer. Therefore, the V_d is the volume of the spherical droplet ($V_d = \frac{\pi}{6} {d_d}^3$). Q_w is assumed to be the atomization rate of precursors, which was calculated at different levels of nebulization volume. Q_g was 8 L/min according to the experimental conditions. The results of these calculations are summarized in **Table S2**.

Table S2. The number of cores in different nebulizing levels.

Level of Nebulizing volume level [-]	Duty cycle* [%]	Atomization rate** [mL/s]	Number of cores [cores/cm^3 carrier gas]
1	30	0.00292	0.43×10^6
2	38	0.00369	0.54×10^6
3	45	0.00438	0.64×10^6
4	53	0.00515	0.76×10^6
6	69	0.00671	0.99×10^6
7	77	0.00749***	1.10×10^6

* Duty cycle is determined by the linear equation, 0.0778x + 0.2222

** The atomization rate is calculated by multiplying the duty cycle by the maximum rate of atomization

*** The maximum atomization rate is determined from the experiment.

3. Effect of Increasing the Degree of Reduction with an Excessive Number of Cores on the Crystal Structure

Changing the ethanol concentration increased the degree of reduction during synthesis. In the case of an excessive number of cores available (1.10×10^6 cores/cm^3 carrier gas), the α-Fe peak significantly improved, and the FeO peak decreased when the ethanol concentration increased from 25% to 30% (v/v), as shown in **Fig. S2**. This improvement is attributed to the extent of the reduction in FeO conversion to α-Fe.

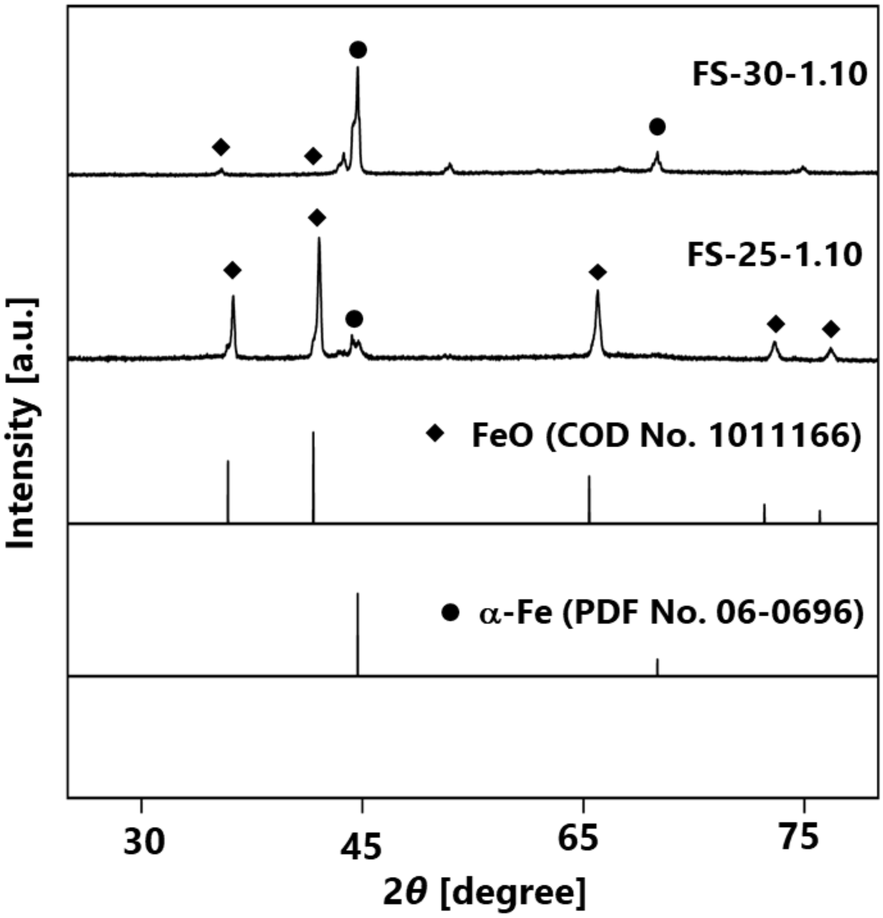

Fig S2. XRD pattern of Fe@SiO_2 particles with an excessive number of cores at different reduction degrees.

4. Determining the Ratio of Number of SiO_2 Monomer per Core Particle

The ratio of number of SiO_2 monomer per core particle was calculated by dividing the number of SiO_2 monomer by the number of core particle. The value of the number of core particles was the same as that of number of cores, as shown in **Section 2**, assuming that all droplets were converted to particles. The number of SiO_2 monomers is defined as the number of supplied SiO_2 in this system. The Antoine equation approximation is used for calculating the supplied SiO_2 from hexamethyldisiloxane (HMDSO). Firstly, the vapor pressure of HMDSO was calculated using the Antoine equation (Equation (2)).

$$\log 10\, P_{v\,(HMDSO)} = A - \frac{B}{T_{HMDSO} + C} \qquad (2)$$

Where P_v is the vapor pressure of HMDSO in mmHg; T_{HMDSO} is the temperature of HMDSO in the bubbler; and A, B, and C are constant properties of HMDSO (CAS No 107-46-0). The partial pressure of HMDSO (x_p) can be calculated based on the vapor pressure of HMDSO. Moreover, the HMDSO volumetric flow rate, $Q_{v(HMDSO)}$, could be determined based on the flow rate HMDSO vapor (Q_s = 10 mL/min) using the equations (3) and (4):

$$x_p = \frac{P_{v(HMDSO)}}{P^0} \qquad (3)$$

$$x_p = \frac{Q_{v(HMDSO)}}{Q_{v(HMDSO)} + Q_s} \qquad (4)$$

Using the HMDSO volumetric flow rate, the number of moles of HMDSO could be calculated using the ideal gas equation (PV = nRT). The number of moles of silica supplied here was twice that of the HMDSO. The mass of the supplied SiO_2 (m_{SiO_2}) was calculated by multiplying the number of moles by the molecular weight, assuming that all HMDSO were converted to SiO_2. The volumetric rate of the supplied SiO_2 (V_s) was determined by dividing m_{SiO_2} by the SiO_2 density. The volumetric rate of supplied SiO_2 was 5.56×10^{-6} cm^3/s. The

number of the supplied silica is determined by dividing the V_s and volume of silica monomer (V_m). Here, V_m was assumed to be the same as the volume of the SiO_2 molecule (4.53×10^{-23} cm^3).[10]

5. Rietveld Refinement Result of Fe@SiO_2 Particle at Different Number of Core

Table S3. Rietveld analysis result of Fe@SiO_2 particles with different numbers of cores.

Name	*R* factor		GOF	Weight percentage [wt%]	
	R_{wp}	R_{exp}		α-Fe	FeO
FS-25-1.10	9.71	5.58	1.71	13	87
FS-25-0.64	13.21	5.36	1.36	56	32
FS-30-0.43	14.63	5.13	2.35	86	14
FS-30-0.54	13.65	5.40	2.53	95	5
FS-30-0.64	10.38	16.58	1.87	100	0
FS-30-0.76	12.31	5.40	2.28	99	1
FS-30-0.99	9.56	5.42	1.76	97	3
FS-30-1.10	12.71	5.39	2.36	96	4

6. Calculation of the Ratio of Mole of Supplied H_2 per Mole Core Particle

The supplied H_2 encompasses the total H_2 utilized in the reaction, including the H_2 derived from the carrier and additional gases as well as the H_2 generated through ethanol conversion. To determine supplied H_2 from the carrier gas (Q_c) and additional gas (Q_a), we employed an H_2 gas mixture with a composition of 5% H_2/Ar, which was multiplied by the total volumetric gas flow rate. The H_2 consumed was converted into moles by multiplying it by the gas density. The density of the 5% H_2/Ar gas was 1.696 kg/m^3 (Data sheet NL-PIB-0284/04). The supplied H_2 was calculated to be 13.3 moles/h, whereas that of the additional gas was 8 moles/h. Furthermore, the H_2 generated from ethanol conversion was determined by stoichiometry. The ethanol conversion equation[11] is shown in Equation (5):

$$C_2H_5OH + 3H_2O \rightarrow 2CO_2 + 6H_2 \tag{5}$$

Based on the use of 30% (v/v) ethanol in this study, the generation of H_2 is 2.1 moles/h. Furthermore, we determined the ratio of moles of supplied H_2 per moles of core particle. The latter was calculated based on the stoichiometry of the mole of precursors according to Equations (6)–(9):

$$2\,Fe(NO_3)_3 \cdot 9H_2O \rightarrow Fe(OH)_2 \cdot NO_3 + H_2O + HNO_3 \rightarrow Fe_2O_3 + NO_2 + H_2O \tag{6}$$

$$3Fe_2O_3 + H_2 \rightarrow 2Fe_3O_4 + H_2O \tag{7}$$

$$2Fe_3O_4 + 2H_2 \rightarrow 6FeO + 2H_2O \tag{8}$$

$$FeO + H_2 \rightarrow Fe + H_2O \tag{9}$$

The number of mole of precursors was determined by multiplying the concentration of precursors by their volume and then dividing by the volume of the particles. Therefore, the number of moles of the core particle was calculated by multiplying the stoichiometrically calculated mole of Fe by the number of core particles. The resulting ratio of the molar concentration of supplied H_2 per mole of core particle is shown in **Table S4**.

Table S4. The ratio of the mole of supplied H_2 per mole core particle using 30 vol% (v/v) ethanol

Nebulization volume level [-]	Number of cores [cores/cm^3 carrier gas]	Ratio of mole concentration of supplied H_2 per mole core particle* [moles of H_2/moles of core particle]
1	0.43×10^6	200
2	0.54×10^6	158
3	0.64×10^6	133
4	0.76×10^6	113
6	0.99×10^6	87
7	1.10×10^6	78

* Ratio of mole concentration of supplied H_2 per mole core particle is calculated by dividing mole of total H_2 supplied and mole of core particle**.

** Mole of core is determined by multiplying the mole of Fe and the number of core particle.

7. Effect of Different Degrees of Reduction with Sufficient Number of Cores in the Crystal Structure

To confirm that increasing the concentration of ethanol (as the reduction agent) from 25% to 30% (v/v) with a sufficient number of cores can completely convert FeO to α-Fe, XRD analysis was conducted. As shown in **Fig. S3**, the FeO peak was detected in the **FS-25-0.64** sample when the ethanol concentration was 25% (v/v). Meanwhile, by increasing ethanol concentration to 30% (v/v), the α-Fe peak was obtained in the absence of FeO.

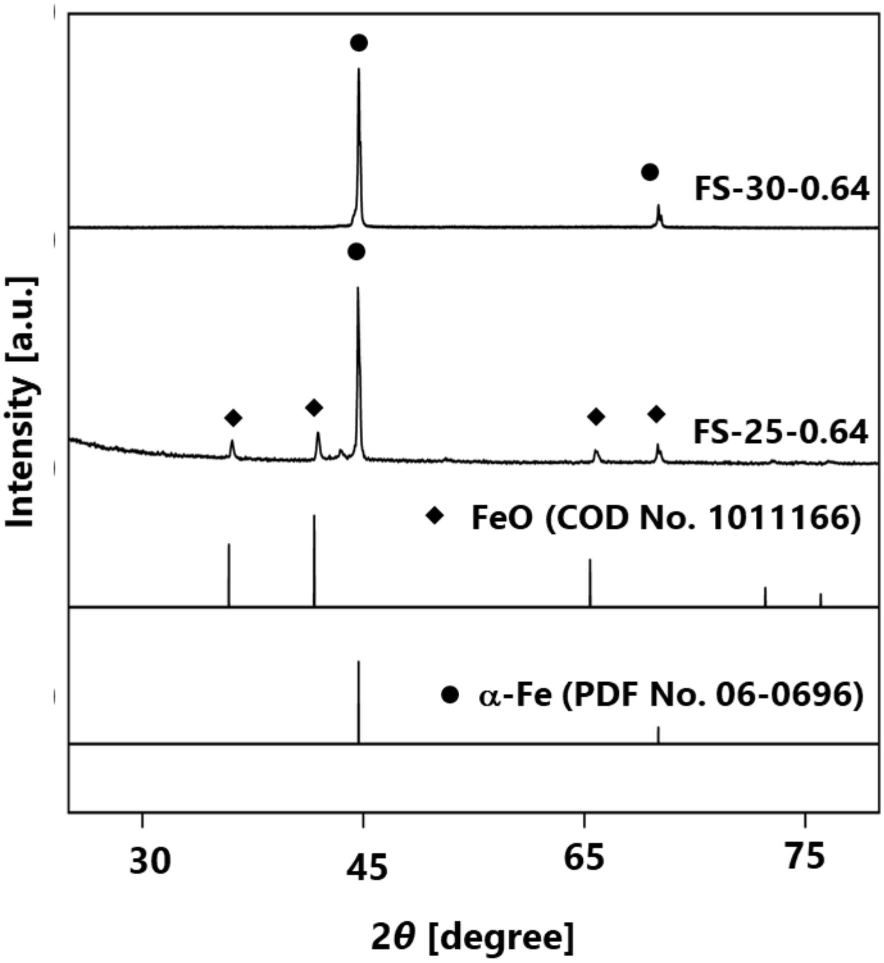

Fig. S3. The X-ray diffraction pattern of $Fe@SiO_2$ particles with a sufficient number of cores at different degrees of reduction.

References

1 W. Zeng, Q. Yang, B. Shao, D. Guo, C. Li, Y. Ma, X. Yin, S. Zhao and K. Li, *Micro Nano Lett*, 2019, **14**, 976–979.

2 X. Ni, Z. Zheng, X. Hu and X. Xiao, *J Colloid Interface Sci*, 2010, **341**, 18–22.

3 A. Zeleňáková, V. Zeleňák, I. Mat'Ko, M. Strečková, P. Hrubovčák and J. Kováč, *J Appl Phys*, 2014, **116**, 033907.

4 Y. Janu, V. S. Chauhan, D. Chaudhary, L. Saini and M. K. Patra, *IEEE Trans Magn*,2019, **55**,1–7.

5 J. Cheng, X. Ni, H. Zheng, B. Li, X. Zhang and D. Zhang, *Mater Res Bull*, 2006, **41**, 1424–1429.

6 G. Kastrinaki, S. Lorentzou, G. Karagiannakis, M. Rattenbury, J. Woodhead and A. G. Konstandopoulos, *J Aerosol Sci*, 2018, **115**, 96–107.

7 S. Stopic, A. H. Hounsinou, K. A. Stéphane, T. Volkov Husovic, E. Emil-Kaya and B. Friedrich, *Metals*,2023, **13**, 1686.

8 W. N. Wang, A. Purwanto, I. W. Lenggoro, K. Okuyama, H. Chang and H. D. Jang, *Ind Eng Chem Res*, 2008, **47**, 1650–1659.

9 Ltd. Omron Healthcare Co., 2015, 23.

10 S. Tsantilis and S. E. Pratsinis, *Langmuir*, 2004, **20**, 5933–5939.

11 E. L. Septiani, J. Kikkawa, K. L. A. Cao, T. Hirano, N. Okuda, H. Matsumoto, Y. Enokido and T. Ogi, *Advanced Powder Technology*, 2021, **32**, 4263–4272.
